# The Critical Role of Adipocytes in Leukemia

**DOI:** 10.3390/biology14060624

**Published:** 2025-05-28

**Authors:** Romane Higos, Kevin Saitoski, Mathieu Hautefeuille, Geneviève Marcelin, Karine Clément, Nadine Varin-Blank, Christophe Breton, Simon Lecoutre, Mélanie Lambert

**Affiliations:** 1 Nutrition and Obesities: Systemic Approach Research Group, Nutriomics, Sorbonne Université, Institut National de la Santé et de la Recherche Médicale (INSERM), 75013 Paris, France; romane.higos@inserm.fr (R.H.); genevieve.marcelin@inserm.fr (G.M.); karine.clement@inserm.fr (K.C.); simon.lecoutre@inserm.fr (S.L.); 2 U1349, Institut National de la Santé et de la Recherche Médicale, 93017 Bobigny, France; kevin.saitoski@inserm.fr (K.S.); nadine.varin@inserm.fr (N.V.-B.); 3 Signalisation Microenvironnement et HEmopathies Lymphoïdes B (SIMHEL), Université Sorbonne Paris Nord, Alliance Sorbonne Paris Cité, 93017 Bobigny, France; 4 Laboratoire de Biologie du Développement (UMR 7622), Institut de Biologie Paris-Seine (IBPS), Sorbonne Université, 75005 Paris, France; mathieu.hautefeuille@sorbonne-universite.fr; 5 Department of Nutrition, Pitie-Salpêtriere Hospital, Assistance Publique-Hôpitaux de Paris, 75013 Paris, France; 6 U1283-UMR8199- European Genomic Institute for Diabetes (EGID), Université de Lille, INSERM, Centre National de la Recherche Scientifique (CNRS), Centre Hospitalier Universitaire (CHU) Lille, Institut Pasteur de Lille, 59000 Lille, France

**Keywords:** leukemia, adipocyte, bone marrow

## Abstract

The bone marrow microenvironment is a dynamic and complex niche that plays a central role in the development, progression, and therapeutic resistance of leukemia. Among the various stromal and immune cells that compose this microenvironment, adipocytes are increasingly recognized as active participants rather than passive bystanders. These cells contribute to leukemia pathophysiology by supplying leukemic cells with vital metabolic fuels such as free fatty acids and glutamine, which support cellular bioenergetics and biosynthesis. Furthermore, adipocytes secrete adipokines—including leptin, adiponectin, and others—that influence leukemic cell proliferation, apoptosis, and chemoresistance. Leukemic cells, in turn, are not merely recipients of these signals, but actively remodel the marrow niche to their advantage. They can suppress adipogenesis, inhibit the differentiation of mesenchymal stem cells into adipocytes, or reprogram existing adipocytes to adopt a tumor-supportive phenotype. These transformed adipocytes may enhance leukemic cell survival, dampen immune responses, and create a metabolic sanctuary that enables resistance to standard chemotherapies. This reciprocal and dynamic interaction between leukemic cells and adipocytes contributes significantly to minimal residual disease and relapse, posing a major challenge for durable remission. Recent advances in tissue engineering—such as organ-on-chip and 3D co-culture systems—offer promising platforms to recapitulate and study these leukemia–adipocyte interactions with high fidelity. These models facilitate mechanistic insights and provide a foundation for developing novel therapeutic strategies aimed at disrupting the metabolic and paracrine crosstalk within the leukemic niche. Targeting the adipocyte–leukemia axis represents a compelling and underexplored avenue for improving leukemia treatment by sensitizing malignant cells to existing therapies and overcoming the protective influence of the bone marrow microenvironment.

## Simple Summary

This review explores how leukemia thrives in the bone marrow, where adipocytes supply vital nutrients and send signals that support cancer growth. In response, leukemia cells hijack these adipocytes, reshaping them to boost their survival, evade treatment, and undergo fuel relapse.

## 1. Introduction

A growing body of evidence highlights the critical role of the cancer microenvironment in driving tumor progression, metastasis, and response to therapy [1,2]. This intricate microenvironment is composed of cancer cells, surrounding normal cells, and the extracellular matrix, along with the signaling molecules that mediate their interactions. In solid tumors, cancer cells engage with a diverse array of host cells, including fibroblasts, macrophages, lymphocytes, endothelial cells, and adipocytes [2,3]. This transformation enhances nutrient availability, shields cancer cells from immune surveillance, and supplies growth factors and survival signals. Collectively, these adaptations enable cancer cells to thrive, proliferate, and metastasize [2,3]. While solid tumors rely heavily on localized interactions within their microenvironment, leukemic cells present a distinct challenge due to their systemic dissemination and ability to colonize multiple niches. These malignant cells traverse and exploit various physiological landscapes, including the bone marrow (BM), spleen, lymphatic system, intravascular compartments, and other extramedullary tissues. Of these, the BM emerges as a central hub for leukemia pathophysiology—serving not only as the primary site of leukemic cell expansion but also as a main sanctuary for disease persistence. Deciphering the intricate interplay between leukemic cells and the BM microenvironment is therefore crucial for advancing therapeutic strategies and disrupting the mechanisms that underpin disease progression.

Bone marrow is a dynamic and heterogeneous tissue found primarily within the medullary canals of long bones such as the tibia, femur, and humerus, as well as in the vertebrae and iliac crest. It serves as the principal site for the differentiation of hematopoietic and mesenchymal cell lineages [4]. BM orchestrates a delicate interplay of cellular processes that begin with pluripotent stem cells and culminate in the production of mature, functional cells vital for homeostasis. This intricate microenvironment comprises a diverse assembly of cell types. Hematopoietic progenitors fuel the production of erythrocytes, megakaryocytes, and immune cells like B and T lymphocytes, natural killer cells, monocytes, and phagocytes [5,6]. Meanwhile, bone-residing osteoblasts, osteocytes, and osteoclasts perpetuate the balance between bone formation and resorption [7]. However, beyond these well-studied players lies an equally significant but often overlooked component: bone marrow adipose tissue (BMAT).

BMAT occupies a substantial portion of the BM cavity—50–70% in lean, healthy adults—and accounts for over 10% of total body fat [8,9,10]. From birth, BMAT begins its exponential accumulation, with distal bones experiencing more rapid adipogenesis than proximal ones. By the age of 25, approximately 70% of human BM consists of BMAT [11]. Longitudinal studies tracking individuals from infancy to advanced age reveal that even after the stabilization of the mature BMAT phenotype, gradual accumulation persists throughout life [9,12,13]. BMAT have also an immunoregulatory function. Through the secretion of proinflammatory cytokines, BMAT contributes to a low-grade inflammation known as “*inflamm-aging*” [14]. These findings underscore BMAT’s dynamic and context-dependent role in bone health and its broader implications for skeletal, immunity, and metabolic physiology.

BMAT has also been increasingly implicated in hematological malignancies. It has been shown to support tumor cell proliferation in leukemia, with niche adipocytes playing a pivotal role in protecting cancer cells from chemotherapy, thereby contributing to treatment resistance [15,16,17,18]. Following induction of chemotherapy for leukemia, adipocytes often become the predominant cellular component in the BM [19]. This striking transformation underscores their significant influence on cancer cell behavior and disease progression, necessitating further investigation into their role in shaping the tumor microenvironment.

The interplay between the BM microenvironment, adipocytes, and leukemic cells highlights the critical need for continued research into these interactions. By unraveling the mechanisms that govern these relationships, researchers may unlock novel therapeutic strategies, offering promising avenues for improved outcomes in both solid tumors and hematological malignancies.

## 2. What Is Bone Marrow Adipocyte?

Adipose tissue plays a vital role in systemic energy balance and comes in different forms, each with unique functions. The two primary types are white adipose tissue (WAT), which primarily stores energy and regulates endocrine function, and brown adipose tissue (BAT), known for its thermogenic ability [20]. Interestingly, cold exposure can transform WAT by inducing the formation of beige adipocytes, enhancing metabolic activity. Both brown and beige adipocytes contain numerous small, multilocular lipid droplets and high mitochondrial density, enabling greater energy expenditure [20]. This energy-burning process is mainly driven by uncoupling protein 1 (UCP1), which disrupts the proton gradient across the inner mitochondrial membrane, separating respiration from ATP production [21]. While WAT, BAT, and beige fat are well characterized, BMAT remains a largely unexplored frontier in metabolic and skeletal biology.

BMAT begins to develop as early as birth, primarily appearing in distal skeletal regions [22,23] and gradually expanding throughout life within hematopoietic marrow [11]. Unlike WAT and BAT, which fluctuate with energy demands, BMAT accumulates persistently, increasing with age and under various physiological and pathological conditions such as obesity, type 2 diabetes, osteoporosis, radiotherapy, glucocorticoid exposure, and caloric restriction [10,24]. Notably, the activation of Peroxisome Proliferator-Activated Receptors γ (PPARγ)—a master regulator of adipogenesis that is necessary for adipocyte differentiation—drives significant BMAT expansion, often at the cost of bone density, as evidenced by PPARγ agonist rosiglitazone treatment [25]. Structurally, BMAT adipocytes (BMAs) resemble WAT cells, forming from preadipocytes that accumulate lipids into unilocular droplets, eventually displacing the nucleus and cytoplasm to the periphery [26].

***Developmental Origins and Lineage Relationships:*** One of the central questions in BMAT biology is its developmental trajectory and lineage relationship to other mesenchymal cells within the BM. The transcription factor Osterix (Osx1), essential for osteoblast differentiation, plays a crucial role in this process [27]. BMAs originate from BM mesenchymal stromal cells (BM-MSCs), sharing a common lineage with osteoblasts. Advanced lineage-tracing studies using “mT/mG” reporter mice have revealed that, unlike gonadal WAT and intramuscular fat, BMAs are uniformly labeled in Osx1-cre:mT/mG models. This finding suggests that Osx1+ progenitor cells give rise to both osteoblasts and adipocytes within the BM microenvironment [28]. Notably, a subset of BMAs maintains a close spatial association with the endothelium of marrow sinusoids, indicating a potential functional interplay between adipocytes and the vascular niche [26]. Emerging evidence suggests that distinct waves of Osx1+ progenitor cells contribute differentially to embryonic and adult bone formation [29]. Lineage-tracing studies further indicate the presence of independent progenitor pools within the BM, highlighting a complex and heterogeneous origin for mesenchymal-derived cell types. Among these, LEPR+ perisinusoidal cells (marked by the leptin receptor) are significant contributors to the osteo-adipogenic lineage [30], while Gremlin 1+ cells, identified by their expression of the bone morphogenetic protein (BMP) antagonist Gremlin 1, form osteoblasts, chondrocytes, myofibroblasts, and stromal cells but do not differentiate into adipocytes [31]. These findings delineate two distinct progenitor pools within the BM: (1) Osx1+, Nes+, and LEPR+ cells, which generate osteoblasts, stromal cells, and adipocytes, and (2) Gremlin 1+ cells, which contribute to various mesenchymal lineages while excluding adipogenesis. Additionally, a preadipocyte-like population within the bone marrow, termed marrow adipogenic lineage precursor (MALP) cells, has been identified. These cells are marked by adipocyte-specific promoters such as *Adiponectin* (*Adipoq*) and form a vast, interconnected three-dimensional network intimately associated with sinusoidal blood vessels. Strikingly, genetic ablation of MALP cells using a diphtheria toxin fragment A (DTA) allele leads to a dramatic expansion of trabecular bone throughout the marrow cavity, an effect most pronounced in female mice [32].

Collectively, these findings underscore that BMAs are not merely fat-storing cells, but a developmentally and functionally distinct adipocyte subtype. They arise from specialized progenitor pools within the unique BM microenvironment, setting them apart from both white and brown adipocytes.

***BMAT’s Unique Metabolic Profile:*** Despite its structural resemblance to WAT, BMAT exhibits a unique metabolic profile. A leading hypothesis suggests that BMAs primarily act as lipid reservoirs, sequestering ectopic lipids to shield skeletal osteoblasts from lipotoxic damage [33]. Gene expression analyses confirm that BMAs are bona fide fat cells, expressing classic adipogenic regulators such as *Pparg, Cebpa, and Cebpb*—similar to WAT [34]. However, BMAT uniquely enriches cholesterol metabolism-related proteins while downregulating those involved in lipolysis, resulting in monoacylglycerol accumulation and impaired lipolysis [8].

Transcriptomic studies in rabbits and humans further highlight differences in glucose metabolism and insulin responsiveness, with BMAT exhibiting higher basal glucose uptake and altered glucose transporter expression compared to WAT, with increased *SLC2A1* (Glucose transporter 1 (*GLUT1*)) and *SLC2A3* (*GLUT3*) expression and lower *SLC2A4* (*GLUT4*) expression [35]. Complementary findings from PET/CT imaging using [^18^F]FDG and analyses of insulin-induced AKT phosphorylation in rodents demonstrate that BMAT is resistant to both insulin- and cold-stimulated glucose uptake. Moreover, in vivo studies confirm that BMAT is less responsive to insulin-stimulated AKT phosphorylation compared to WAT [35]. However, human data present conflicting results. Tam et al. reported that femoral BMAs are insulin-sensitive and capable of increasing glucose uptake, as indicated by [^18^F]FDG tracer uptake [36]. Additionally, their study found that insulin sensitivity in femoral BMAT was impaired in obese patients with type 2 diabetes mellitus compared to healthy controls, but significantly improved following bariatric surgery. These contrasting results highlight significant variations in BMAT metabolism across species (rodents vs. humans) and anatomical sites (e.g., distal tibia BMAT vs. femoral BMAT). Further research is necessary to unravel these complexities and reconcile species- and location-specific differences in BMAT glucose metabolism and insulin responsiveness.

Additionally, it was demonstrated that BMAT is not cold-responsive, as it does not increase glucose uptake during cold exposure [35,36]. This has led researchers to investigate whether BMAT shares characteristics with BAT. Some studies have reported expression of BAT markers, including *Prdm16, Dio2, FoxC2*, and *Pgc1α*, in whole tibia samples [37]. Moreover, exposure of BMAs to triiodothyronine, a thyroid hormone receptor beta-specific agonist (GC-1), or PPARγ agonists significantly upregulates both BAT markers and WAT (*Adipoq* and *Leptin*) markers [4,37,38]. However, results remain inconsistent. Purified BMAs from aged mice did not consistently express these markers [39], and human BMAs showed no significant enrichment of UCP1 or other brown/beige markers [35]. These findings challenge the notion of BMAT as a BAT-like depot and emphasize the importance of using primary BMAT adipocyte samples in cancer research instead of relying solely on WAT-derived adipocytes.

***Endocrine, Hematopoietic, and Immunoregulatory Roles:*** Beyond energy storage, BMAT is increasingly recognized as an active endocrine organ, secreting adipokines that influence systemic metabolism. One such adipokine, adiponectin, plays a crucial role in insulin sensitivity and energy homeostasis [40]. Unlike WAT, BMAT exhibits elevated adiponectin expression and secretion, particularly during periods of starvation and in patients undergoing cancer therapy [10]. By producing adiponectin, BMA has the potential to exert systemic effects on metabolic homeostasis, immune responses, vascular function, or cancer risk, but the consequences of elevated adiponectin during cancer therapy clearly warrant further investigation [10]. Leptin, another key adipokine, is also produced by BMAs [41], though its regulation differs from that of WAT. Inflammatory cytokines such as IL-1β, IL-6, TNF-α, and IFN-γ significantly suppress *leptin* gene expression in BMAs [42]. Leptin signaling in BM mesenchymal stromal cells promotes adipogenesis while inhibiting osteogenesis through Jak2/Stat3 activation, a mechanism influenced by diet and adiposity [43]. Transcriptomic analyses of human BMAs have revealed a heightened expression of proinflammatory cytokines, notably IL-6, IL-15, and reactive oxygen species (ROS), underscoring a potential immunoregulatory role. Notably, femoral BMAs exhibit higher adipokine secretion than subcutaneous thigh WAT and are capable of suppressing IgG-producing plasma cells [14]. Furthermore, the interplay between BMAT and immune cells appears responsive to nutritional states. During dietary restriction, BMAT contributes to the accumulation of memory T cells in the BM—unlike WAT, which typically supports memory T cell maintenance and reactivation [44].

***A Multifaceted Role in Skeletal and Metabolic Physiology:*** BMAT plays a multifaceted role in both bone integrity and hematopoiesis. Emerging evidence suggests its impact on the mechanical properties of bone, although the precise mechanisms remain poorly understood. Closely integrated with the hematopoietic niche, BMAT’s contribution to blood cell production continues to be a topic of active investigation and debate. In vivo studies in mice indicate that BMAT predominantly suppresses hematopoiesis by inducing progenitor cell quiescence and depletion [45]. This inhibitory role is further substantiated by experiments employing pharmacological inhibition of adipogenesis and genetic manipulation in “*fatless*” A-ZIP/F1 mice, which demonstrated accelerated BM engraftment following irradiation in the absence of BMAT [45]. Moreover, studies have linked increased BMAT mass, as seen during obesity and aging, with impaired hematopoiesis [46,47]. High-fat diets in mice have also been shown to promote BMAT accumulation, accompanied by an expansion of myeloid lineages at the expense of lymphopoiesis [48]. Nevertheless, this relationship is complex and often contradictory. In vitro experiments reveal that primary human BMA, isolated from the iliac crest, support the differentiation of CD34+ hematopoietic progenitors [49]. Furthermore, PPARγ agonist rosiglitazone-induced BMAT expansion in mice did not significantly affect hematopoietic progenitor frequencies [50]. Conversely, prolonged exposure to low temperatures reduces BMAT content [34] and is associated with a decline in myeloid cell counts in hibernating mammals [51]. These findings underscore the intricate and context-dependent nature of the adipogenesis–hematopoiesis axis, revealing it as a dynamic and underexplored area of biological interplay.

BMAT stands at the intersection of skeletal and metabolic physiology, embodying both structural and endocrine functions that remain incompletely understood. Its dual role as an energy reservoir and a regulator of BM niches underscores its complexity. Future research is essential to uncover the precise molecular mechanisms governing BMAT function and its implications for metabolic and bone diseases.

## 3. Adipocytes: Silent Partners in Leukemia’s Journey

Bone marrow adipocytes are no longer mere bystanders in the BM niche, but are now recognized as active participants in leukemia progression and therapy response. Bone marrow adipocytes undergo profound changes during leukemia pathogenesis and treatment, shifting from cellular depletion in the primary leukemia niche to a reconstituted state during remission. Emerging evidence paints a vivid picture of leukemic cells co-opting BMAs to shield themselves from chemotherapy-induced cytotoxicity through diverse mechanisms. This realization has sparked a novel paradigm: targeting the leukemia–adipocyte interaction as a therapeutic strategy [19,52,53].

Similar to subcutaneous and visceral WAT [40], BMAs undergo remodeling in response to metabolic and mechanical cues, including changes in size, immune infiltration, fibrosis, and vascular remodeling. While peripheral WAT expansion is primarily an adaptive mechanism for energy storage, BMAs contribute to the regulation of hematopoietic stem cells (HSCs) and the leukemic niche. In leukemia, BMAs have been shown to undergo reprogramming, supporting leukemic cell survival by secreting cytokines such as stromal cell-derived factor 1 (SDF-1 also known as CXC motif chemokine 12 (CXCL12)), which facilitates the homing and retention of leukemic cells in the BM [54].

***SDF-1: A Central Mediator in the Marrow Niche:*** Recent studies highlight the crucial role of SDF-1 expression in BMAT, demonstrating its impact on skeletal homeostasis, metabolic regulation, and hematopoietic niche function. The deletion of Cxcl12 in mesenchymal progenitor (particularly in Prrx1+ cells, which target early limb bud and some craniofacial mesenchyme) results in increased marrow adiposity and erythropoiesis, alongside a dramatic 90% reduction in B lymphocytes and a loss of trabecular bone [55]. In WAT, Cxcl12 is also expressed by mature adipocytes, where both fasting and obesity induce its upregulation. Cxcl12 acts in an autocrine fashion to activate extracellular signal-regulated kinases (ERK) signaling, which triggers serine phosphorylation of insulin receptor substrate 1 (IRS-1), resulting in IRS-1 degradation. This cascade ultimately impairs insulin-mediated Akt phosphorylation and glucose uptake, contributing to insulin resistance in WAT adipocytes [56]. However, despite these insights from WAT, the expression of Cxcl12 by BMAs remains poorly characterized. Determining how BMAs produce Cxcl12—and understanding the conditions that regulate this expression—would be a major step forward in clarifying their role in the BM microenvironment [32]. If confirmed, Cxcl12-producing BMAs could emerge as powerful regulators of hematopoiesis, immune cell development, and bone remodeling.

Interestingly, both caloric restriction and leptin signaling influence Cxcl12 levels in BM-derived mesenchymal stem cells, indicating a potential link between nutrient availability and mesenchymal differentiation [57]. Moreover, silencing of connective tissue growth factor (CTGF) in MSCs leads to heightened adipogenesis and increased Cxcl12 production, potentially fostering an environment conducive to leukemic cell engraftment [58]. This CXCL12/CXCR4 axis also holds promise for adoptive immunotherapy. Targeting this pathway could enhance the homing of genetically engineered natural killer (NK) cells to leukemic niches, offering innovative avenues for improving leukemia clearance and advancing hematologic cancer treatments [59,60].

***The Leukemic Remodeling of the Marrow Adipocyte Niche:*** In B cell acute lymphoblastic leukemia (B-ALL), the BMA niche undergoes dynamic remodeling. At diagnosis, there is a stark depletion in adipocyte numbers and size, reflecting severe suppression of adipogenesis. Yet, as remission is achieved, adipocyte populations recover, evidencing a return to homeostasis [61]. In contrast, acute myeloid leukemia (AML) demonstrates persistent adipogenic impairment during chemotherapy. Perilipin immunohistochemistry reveals significantly smaller and fewer BMAs in AML patients compared to healthy controls [62,63]. Interestingly, smaller adipocytes have been linked to worse prognoses in AML patients [63]. Functional changes further underscore these disruptions; adiponectin, a key marker of BM adiposity, is significantly reduced in the marrow plasma of ALL patients [62,64].

Leukemic cells are known to actively manipulate the BMAT. Through transforming growth factor β (TGF-β) secretion, they inhibit the adipogenic differentiation of BM mesenchymal stem cells (BM-MSCs), suppressing the production of mature adipocytes [65,66]. However, this disruption is not without therapeutic countermeasures. PPARγ agonists have been shown to promote adipogenesis in BM, restoring healthy hematopoiesis while concurrently suppressing leukemic growth [65].

**Figure 1:**
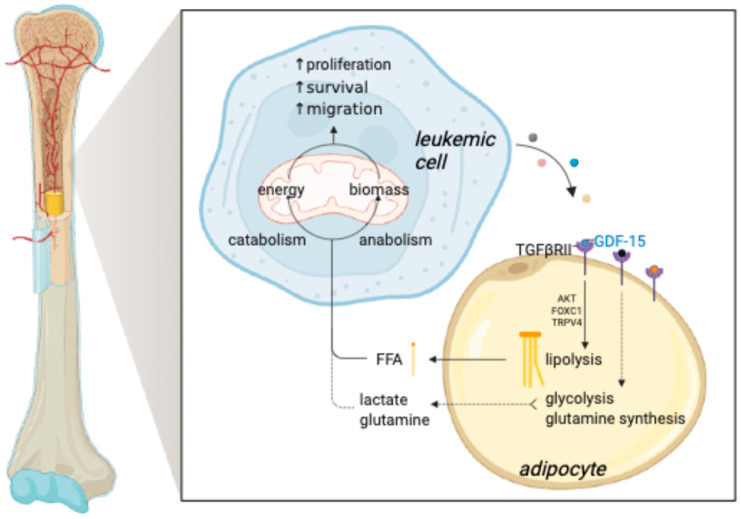
Metabolic interactions between BMAs and leukemic cells. Leukemic cells orchestrate a dynamic metabolic crosstalk with BMAs by secreting GDF15, which binds to TGFβRII receptors on adipocytes, initiating the PI3K/AKT signaling cascade. This activation suppresses the transcription factor FOXC1, downregulating TRPV4 expression and driving BMAT adipocyte remodeling. The resulting reduction in Ca^2+^ influx amplifies the expression of ATGL and HSL, key regulators of lipolysis, leading to the breakdown of stored lipids. Consequently, BMAs release free fatty acids, which leukemic cells exploit as an energy source or as building blocks to fuel their expansion. Furthermore, BMAs act as metabolic hubs, supplying leukemic cells with glutamine and lactate—critical nutrients that sustain tumor growth and survival. Illustration created with BioRender (OJ287VL91L).

A key player in AML’s interaction with BMAT is growth differentiation factor 15 (GDF15). Leukemic cells secrete GDF15, remodeling residual adipocytes into smaller, dysfunctional forms, a process associated with poor prognosis. GDF15 acts through the TGF-β type II receptor (TGFβRII) on BMA, activating downstream signaling pathways like PI3K and AKT. Downstream activation of this pathway suppresses the transcription factor FOXC1, thereby inhibiting the expression of transient receptor potential vanilloid 4 (TRPV4)—a calcium-permeable ion channel critical for adipocyte homeostasis. Consequently, downregulation of TRPV4 may lower intracellular calcium levels, which in turn might enhance hormone-sensitive lipase (HSL) activity, promoting adipocyte lipolysis. The resulting release of free fatty acids (FFAs) could then be hijacked by leukemic cells as metabolic substrates to sustain their growth and proliferation [67]. AML blasts further exploit BMAT by inducing triglyceride hydrolysis, providing essential fatty acids for their growth and survival [68]. These studies reinforce the idea that leukemic cells ingeniously manipulate their surroundings, enlisting adipocytes through intricate mechanisms to fuel their own progression (Figure 1).

***Adipocyte Influence on Leukemia: A Double-Edged Sword:*** The interplay between leukemic cells and adipocytes reveals both supportive and inhibitory roles, depending on the leukemia type and niche context. For instance, while AML thrives on adipocyte depletion [65], ALL proliferation can be suppressed by adipocytes, as demonstrated in co-culture experiments with 3T3-L1 cells and MSC-derived adipocytes [69]. BMAT’s effects appear lineage-specific; in T-ALL, adipocytes inhibit proliferation but paradoxically promote chemoresistance. This is achieved by driving leukemic cells into quiescence, a slow-proliferating state that is less vulnerable to chemotherapy. Mechanistically, adipocyte interactions suppress protein biosynthesis in ALL cells via post-transcriptional regulation. Treatment with general control nonderepressible 2 (GCN2) inhibitors (GCN2ib) reverses this suppression, reducing adipocyte-mediated protection and improving chemotherapy efficacy [69,70]. Thus, adipocytes shield leukemic cells from the cytotoxic effects of frontline therapies. Behan et al. explored this relationship using pre-B ALL cell transplants in mice fed a high-fat diet or normal diet [71]. While obesity did not accelerate leukemia progression, it significantly reduced the efficacy of vincristine, a key chemotherapeutic agent. Adipocytes further blunted the effects of dexamethasone, daunorubicin, and nilotinib on both murine and human ALL cells [71,72]. However, the story takes a surprising turn with L-asparaginase (ASNase), a first-line ALL treatment. ASNase depletes asparagine and glutamine, starving leukemic cells. Yet, BMAs counteract this by ramping up glutamine synthetase production post-chemotherapy, neutralizing ASNase’s effects [73].

This intricate dance between leukemic cells and BMAT underscores the complexity of the BM microenvironment. Adipocytes, once overlooked, emerge as critical players in leukemia pathogenesis, offering both challenges and opportunities for therapeutic intervention. By unraveling these interactions, researchers can pave the way for novel treatments that target the leukemia–adipocyte axis, reshaping the battlefield in the fight against hematologic malignancies.

## 4. Adipocytes Support Leukemia Survival and Resistance by Supplying Metabolites

The pivotal role of metabolism in cancer was unveiled nearly a century ago by Otto Warburg, whose discovery of aerobic glycolysis—now famously known as the Warburg effect—revealed cancer cells’ unique ability to convert glucose into lactate, even in the presence of oxygen [74]. This metabolic strategy equips tumor cells with both energy and molecular precursors to fuel their relentless proliferation. Today, dysregulated metabolism is firmly established as a hallmark of cancer [75]. Although targeting cancer metabolism has long tantalized researchers, the road to clinical translation remains fraught with challenges, including limited efficacy in vivo and unintended toxicities, such as hemolysis and compromised immune cell function [76].

***Metabolic Flexibility in Leukemia*:** In leukemia, metabolic plasticity is particularly pronounced. Leukemic cells exhibit a profound upregulation of key metabolic circuits to satisfy heightened energetic and biosynthetic demands. Studies from both experimental models and patient samples have consistently highlighted glycolysis as a cornerstone of leukemic metabolism, particularly in AML and ALL [77]. In the widely used AML MLL-AF9 mouse model, adenosine monophosphate-activated protein kinase (AMPK) was found to suppress leukemic cell function by curbing glycolysis and elevating oxidative stress [77]. AMPK is one of the main sensors of cellular energy status in effectively all eukaryotic cells, and is activated in response to energy stress by sensing increases in AMP:ATP and ADP:ATP ratios and restores energy balance by inhibiting anabolic processes that consume ATP, while promoting catabolic processes that generate ATP [78]. Its deletion delays leukemogenesis, reduces leukemia-initiating cells, downregulates Glut1, and heightens oxidative damage. Intriguingly, dietary restriction, which activates AMPK, preserves leukemic potential—underscoring the metabolic adaptability of leukemic cells [77]. AML blasts rely heavily on glycolysis, and glucose deprivation significantly impairs their viability in vitro [79]. Yet, the story is more complex. CLL cells, despite exhibiting reduced glucose uptake relative to normal B cells [80,81], continue to engage in active glycolysis and maintain robust lactate production [82,83]. Moreover, tonic activation of the B-cell receptor in lymphoma cell lines has also been linked to glycolytic activation [84]. Encouragingly, inhibitors of glucose uptake, such as ritonavir, and glycolysis inhibitors like 2-deoxy-D-glucose (2DG) have demonstrated in vitro cytotoxic effects on CLL cells [85,86]. Beyond glycolysis, cancer cells harness additional metabolic pathways, including glutamine metabolism, to sustain their energy needs [87]. Unlike glycolysis, oxidative phosphorylation (OXPHOS) derives energy from the catabolism of diverse metabolites, including amino acids, fatty acids, and glucose, which feed into the tricarboxylic acid (TCA) cycle. Recent studies have illuminated the differential use of these metabolites in generating TCA intermediates [79,88], revealing yet another dimension of metabolic flexibility in cancer.

***Metabolites as Signaling Molecules:*** Metabolites are far more than mere energy sources; they act as powerful signaling molecules capable of reshaping the cellular landscape. Changes in specific metabolic intermediates can drive profound shifts in gene and protein expression by influencing epigenetic modifications on DNA, RNA, and histones or even by directly modulating protein synthesis. These metabolic signals are not confined to cancer cells alone [75]. As a tumor grows, the altered composition of its surrounding metabolites orchestrates the behavior of neighboring non-cancerous cells such as adipocytes, fueling further tumor expansion. But the influence of tumors extends even further. Emerging evidence reveals that tumors engage in a systemic metabolic crosstalk, impacting the metabolic balance of the entire organism. In leukemia, this metabolic reprogramming plays a pivotal role in disease initiation. Mutations in isocitrate dehydrogenase isoforms IDH1 and IDH2 drive the production of the oncometabolite D-2-hydroxyglutarate (D-2HG), which inhibits key epigenetic regulators such as histone demethylases and the TET family of 5-methylcytosine hydroxylases. This disruption leads to persistent histone and DNA methylation, blocking cellular differentiation and setting the stage for leukemia development [89,90]. These findings highlight the far-reaching impact of metabolic reprogramming in leukemia, underscoring its critical role in shaping both the tumor microenvironment and the systemic “*metabolic economy*”.

***Adipocytes Fueling Leukemia:*** Adipocytes, traditionally viewed as mere energy reservoirs, have emerged as pivotal players in leukemia progression. Their primary role—storing energy in the form of triglycerides—becomes subverted in the leukemic niche. Leukemic cells reprogram adipocytes, triggering lipolysis and facilitating the transfer of FFAs to AML blasts via the fatty acid-binding protein 4 (FABP4) pathway [68]. FABP4, highly expressed in adipose tissue, functions as a lipid chaperone, and its transcription is upregulated in both adipocytes and leukemic blasts in co-culture settings [91]. Knockdown of FABP4 using shRNA significantly impaired AML proliferation and improved survival in a Hoxa9/Meis1-driven AML model [68]. AML mitochondria leverage these FFAs as substrates for β-oxidation, fueling leukemic cell growth [92]. Increased FFA levels further exacerbate leukemia-associated inflammation and enhance leukemic cell oxidative metabolism [93].

In CLL, lipid metabolism is similarly rewired. CLL cells aberrantly express lipoprotein lipase (LPL), a key enzyme facilitating lipid uptake and storage, which is typically restricted to adipocytes and muscle cells [94]. High LPL expression correlates with aggressive disease and poor prognosis [83,95], while patients with mutated IgHV exhibit lower LPL levels due to promoter hypermethylation [96,97]. LPL, driven by constitutively active STAT3, promotes lipid storage and metabolic adaptation, enabling CLL cells to preferentially utilize lipids for OXPHOS [83,98]. This lipid-centric metabolic reprogramming provides a compelling rationale for targeting lipid metabolism in CLL, where adipocytes act as suppliers under leukemic control.

Glutamine, the most abundant intracellular amino acid, is another key nutrient for leukemic cells [99]. Essential for nucleotide synthesis, protein biosynthesis, and TCA cycle *anaplerosis*, glutamine serves as a vital fuel source [100]. Once inside the cell, glutamine is converted to glutamate via glutaminase (GLS) [101,102], and then further processed into α-ketoglutarate to sustain mitochondrial respiration [103]. Depleting glutamine impairs ATP production, reduces oxygen consumption, and induces apoptosis in AML cells [104]. Pharmacological inhibition of GLS disrupts mitochondrial respiration and proliferation in AML, while in vivo models confirm the anti-leukemic potential of glutaminase blockade [105,106]. Adipocytes, known glutamine producers, may serve as external glutamine suppliers, shielding leukemic cells from metabolic stress [104,107,108]. In high-risk ALL, BMAs upregulate glutamine synthetase following chemotherapy, thereby conferring resistance to asparaginase-induced cytotoxicity [73] (Figure 1).

In conclusion, leukemia’s metabolic adaptability underscores its formidable resilience. By hijacking glycolysis, OXPHOS, lipid metabolism, and glutamine utilization, leukemic cells construct a dynamic metabolic framework that ensures survival under diverse conditions. The intricate interplay between leukemic cells and their microenvironment—particularly adipocytes—reveals new vulnerabilities that could be exploited therapeutically. As research advances, targeting metabolic dependencies holds promise for reshaping leukemia treatment, but precision is key. The road ahead requires unraveling metabolic heterogeneity and developing strategies that disrupt leukemic metabolism without collateral damage to normal cells.

## 5. Burning Metabolites, Battling Cancer: The Antileukemic Role of Thermogenic Adipocytes

Brown and beige adipocytes are best known for their remarkable ability to act as metabolic sinks, avidly consuming circulating nutrients—such as glucose and fatty acids—to fuel their thermogenic function. This property has garnered significant interest in metabolic research, particularly for its potential in mitigating conditions like hyperglycemia and hyperlipidemia [20]. Yet, an unexpected twist in this metabolic tale has emerged: could the same mechanism that allows BAT to siphon nutrients for heat production also starve cancer cells of their vital energy supply?

Much like activated brown and beige adipocytes, cancer cells exhibit an insatiable hunger for nutrients to sustain their rapid proliferation. Traditional approaches to cancer therapy have long sought to block nutrient uptake pharmacologically, depriving tumors of their metabolic fuel. However, an alternative strategy has surfaced—redirecting these nutrients toward an alternative metabolic sink, effectively outcompeting the tumor for its lifeline. Indeed, cancer cells demonstrate exquisite sensitivity to glucose deprivation, forming the rationale behind low-glycemic diets as a means to slow tumor progression [108]. Inspired by this concept, Seki et al. posed an intriguing question: could activating adipocyte thermogenesis exert an anti-tumor effect simply by creating metabolic competition for circulating glucose [109]?

**Figure 2:**
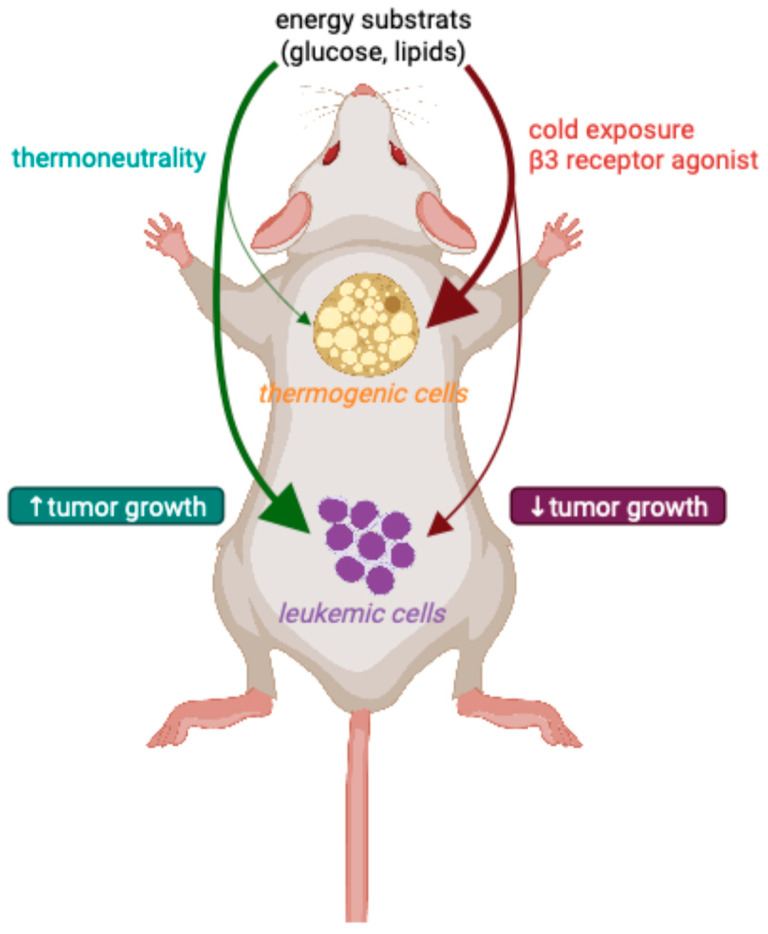
Brown adipose tissue inhibits leukemia progression by siphoning nutrients. Cold exposure inhibits leukemia progression and improves survival in mouse models by activating brown adipose tissue (BAT). This activation, triggered by cold temperatures or β3-adrenergic receptor agonists, enhances nutrient absorption by brown adipocytes, effectively restricting nutrients availability for leukemic cells and suppressing their growth. Illustration created with BioRender (RJ287VLG2H).

Mechanistic investigations revealed that cold-induced activation of adipocyte thermogenesis substantially reduces blood glucose levels, thereby impeding glycolysis-dependent metabolism in cancer cells. The pivotal role of UCP1 was underscored when its genetic deletion abolished the cold-triggered anticancer effects. A pilot study in humans demonstrated that even mild cold exposure can robustly activate BAT in both healthy individuals and a cancer patient—coinciding with a notable reduction in tumor glucose uptake, likely due to increased glucose consumption by activated brown adipocytes, thereby limiting its availability to the tumor [109]. These findings offer an entirely new paradigm for cancer therapy—one that is simple, effective, and physiologically rooted. Beyond cold exposure, pharmacological stimulation of adipocyte thermogenesis has yielded similarly compelling results. Mirabegron, the first clinically available β3-adrenergic receptor agonist approved for the treatment of overactive bladder [110], has been shown to significantly increase adipocyte thermogenesis activity in human subjects, as demonstrated by enhanced uptake of the PET imaging tracer 18F-FDG [111]. Even more strikingly, mirabegron exhibits broad and potent anticancer effects across multiple malignancies. Crucially, its efficacy is entirely dependent on adipose tissue browning; in murine models, both genetic ablation of *Ucp1* and surgical removal of adipose depots nullified its tumor-suppressive effects [112]. These findings highlight adipocyte thermogenesis as a novel mechanism of action for anticancer therapy, distinct from conventional treatments (106,107). Expanding on these discoveries, researchers have now turned their attention to hematological malignancies. In a striking preclinical study, housing mice in cold environments—either before or after leukemic cell transplantation—significantly reduced leukemic cell burden and prolonged survival. Similarly, administration of a β3-adrenergic receptor agonist, known to activate BAT, yielded comparable results. However, when BAT was surgically removed, or *Ucp1* was genetically deleted, the protective effects were completely abolished, confirming that adipocyte thermogenesis plays a pivotal role in suppressing leukemia [109,112,113,114] (Figure 2).

Building upon the premise that adipocyte thermogenesis can be harnessed for cancer therapy, researchers have envisioned an even more ambitious approach: implanting engineered adipocytes designed to outcompete tumors for nutrients. This revolutionary technology—termed Adipose Manipulation Transplantation (AMT)—leverages adipocytes genetically modified to upregulate UCP1, thereby consuming increased amounts of glucose and fatty acids. When placed alongside cancer cells or tumor xenografts, these modified adipocytes effectively suppressed cancer growth. Further demonstrating the promise of AMT, transplantation of engineered adipose organoids into pancreatic and breast cancer mouse models led to profound tumor suppression, coupled with reduced angiogenesis and hypoxia. In human-derived models, co-culturing patient-derived tumor organoids with engineered adipocytes significantly inhibited cancer proliferation. The flexibility of AMT was further illustrated by its ability to target uridine-dependent pancreatic ductal adenocarcinoma by upregulating uridine phosphorylase 1 (UPP1) in adipose organoids—outcompeting the cancer for its essential metabolic substrate and impeding tumor growth [115].

Adipose tissue presents a groundbreaking avenue for cancer therapy, leveraging metabolic mechanisms rather than cytotoxicity. Through cold exposure, pharmacological activation, or cellular engineering, thermogenic brown and beige adipocytes can outcompete tumor cells for nutrients, suppressing cancer progression. This innovative strategy transforms adipocytes into metabolic allies against malignancy. With clinical development, adipocyte-based therapies could offer personalized, non-toxic cancer treatments, akin to CAR-T cell therapy, where engineered cells derived from the patient are reintroduced to combat disease.

## 6. Adipocyte-Derived Adipokines in Leukemia Progression

Adipocytes, long dismissed as passive fat stores, have stepped into the spotlight as cunning collaborators in leukemia’s survival game. Far from being mere bystanders, these lipid-laden cells orchestrate a symphony of signals that influence leukemia’s destiny—balancing cell proliferation and death with surgical precision through a potent brew of adipokines like leptin and adiponectin.

Leptin, the hormone famous for regulating appetite, reveals its darker, insidious side in leukemia. It morphs into an accomplice, bolstering malignant cell survival with anti-apoptotic prowess. Elevated leptin levels in AML patients correlate with enhanced leukemic cell viability [116]. Mechanistically, leptin fortifies primary acute promyelocytic leukemia cells against apoptosis by activating signal transducer and activator of transcription 3 (STAT3) phosphorylation, mitogen-activated protein kinase (MAPK) signaling, and Leptin/LEPR receptor interactions—achieving maximum effect through intimate cell-to-cell contact [117]. Within the BM’s microcosm, leptin secreted by BMAs amplifies leukemic cell proliferation. The evidence? A well-documented link between heightened leptin, suppressed adiponectin, and increased risk of hematologic malignancies. Yet, leptin’s role in CLL is more enigmatic. A Greek study revealed no clear connection between leptin levels and CLL risk, hinting at a complex interplay of factors [118].

**Figure 3:**
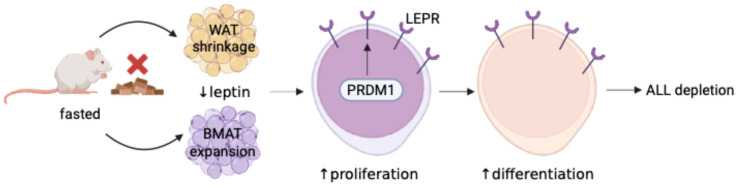
Fasting blocks ALL via leptin-receptor upregulation. Fasting halts the onset and reverses the progression of B and T cell acute lymphoblastic leukemia (B-ALL and T-ALL) in mouse models but not acute myeloid leukemia (AML). Fasting promotes BMAT expansion and lowers plasma leptin levels while enhancing leptin-receptor (LEPR) expression, which is essential for ALL development and maintenance. By activating LEPR signaling via PRDM1, fasting suppresses ALL growth. LEPR-related gene expression correlates with prognosis in pediatric pre-B-ALL, and fasting reduces B-ALL progression in human xenograft models. Illustration created with BioRender (CU287VLLFP).

Known to expand BMAT while suppressing leptin levels [119], fasting emerges as a surprising leukemia disruptor. By upregulating the LEPR and activating its downstream effector, PRDM1—a transcription factor that drives lymphoid progenitor differentiation—fasting forces ALL cells into a state of maturation, effectively halting their proliferation. These malignant blasts, which depend on reduced LEPR expression for survival, find themselves cornered as fasting-induced LEPR signaling dismantles their growth machinery [119] (Figure 3).

The interplay between fasting and leukemia becomes even more striking in the context of obesity. In mouse models, obesity accelerates ALL progression [120] by fostering leptin resistance—a condition marked by elevated leptin levels but weakened LEPR signaling [121]. Within this environment, adipocytes become conspirators, creating a protective niche for ALL cells. High leptin levels in this microenvironment suppress LEPR expression, crippling leukemic cell differentiation and shielding them from chemotherapy’s effects [122]. This phenomenon underscores how adipocytes enable ALL relapse by providing a sanctuary where leptin silences the receptor critical to leukemic cell suppression. Fasting, however, flips this script. By reducing systemic leptin levels, fasting triggers a compensatory rise in LEPR expression on leukemic cells. For ALL cells—uniquely characterized by minimal LEPR expression—this change is transformative. The increase in LEPR levels amplifies leptin signaling despite the overall drop in circulating leptin, driving ALL blasts toward differentiation and undermining their survival. These dynamics contrast sharply with AML cells, which naturally express higher LEPR levels and thus remain unaffected by fasting’s regulatory effects [119] (Figure 3).

While questions linger about fasting’s universal impact on genetically diverse ALL subtypes, these findings illuminate a novel therapeutic avenue. By harnessing fasting-induced LEPR signaling, researchers could reimagine leukemia treatment, turning the metabolic vulnerabilities of ALL into a decisive advantage. What began as a metabolic intervention now stands poised to challenge the very foundations of leukemia’s survival strategies.

In stark contrast to leptin’s malignant influence, adiponectin stands as leukemia’s counterforce, a guardian of cellular balance. Often hailed as a tumor suppressor, adiponectin actively blocks leukemic cell proliferation while inducing apoptosis [123]. Its protective effects ripple through key pathways—AMPK, AKT, MAPK, and GSK3/β-catenin [124,125,126]. By tipping the scales of survival, adiponectin downregulates anti-apoptotic B-cell lymphoma 2 (BCL2) and boosts pro-apoptotic Bcl-2–associated X (Bax), disrupting leukemia’s survival machinery. Notably, it also improves insulin resistance, reinforcing its role as a metabolic powerhouse [127]. Yet, in leukemia’s complex ecosystem, adiponectin’s voice often goes unheard—overshadowed by the sinister dominance of leptin.

But adipocytes wield more than just hormonal messengers. They actively lure leukemic cells into their territory, using CXCL12 as their beacon. This potent chemoattractant, highly expressed in adipose tissue, creates a magnetic pull for leukemic cells, guiding them into protective, adipocyte-rich sanctuaries [56]. CXCL12 binds to CXCR4 receptors on leukemic cells, triggering cascades of cytoskeletal reorganization, integrin activation, and adhesion molecule engagement [128]. Once nestled within these niches, leukemic cells find refuge—shielded from therapeutic assaults and poised to resist.

This intricate dance of adipocyte-derived signals unveils a startling truth: adipocytes are not merely passive participants but active architects of leukemia progression.

## 7. Conclusions

The study of the BM microenvironment has shed light on its pivotal role in shaping leukemia progression and drug resistance. Intracellular signaling within this neoplastic niche facilitates critical cell-to-cell communication, fostering leukemic cell adhesion, growth, and immune evasion. Central to this interplay is the dynamic relationship between adipocytes and leukemic cells, a phenomenon that may reflect evolutionary conservation across mammals.

Animal models have long been indispensable for biomedical research, offering unparalleled insights into disease mechanisms and progression. These systems allow researchers to manipulate genetic and environmental variables with ease and serve as platforms to test drug efficacy in preclinical studies. However, the translation of promising preclinical findings into successful clinical outcomes remains fraught with challenges. The so-called “valley of death” in drug development arises from fundamental species differences in genomics, epigenomics, metabolism, and drug responses, which often render animal models inadequate predictors of human outcomes [129].

Recognizing these limitations, researchers have turned to human cell and tissue-based models. These include 2D immortalized cell lines, patient-derived xenografts, stem cell-derived cell types, and organoids, each of which offers a more relevant framework for studying human disease. Advanced microfluidic systems are particularly promising, enabling the study of intercellular communication between adipocytes and leukemic cells in a controlled environment. Such systems can illuminate how leukemic cells reprogram adipocyte biology, siphoning energy sources like lactate, glutamine, and FFA to fuel their growth and evade chemotherapy.

Organ-on-chip (OoC) models have dramatically improved our ability to mimic the BM microenvironment. Platforms illustrated with statements such as “bone marrow–on–a–chip replicates hematopoietic niche physiology in vitro” [130] and “A multi-niche microvascularized human BM on-a-chip elucidates key roles of the endosteal niche in hBM physiology” [131] have successfully integrated components such as vascular networks, stromal cells, and osteoblastic layers. These systems provide a dynamic environment that closely resembles in vivo conditions, making it possible to study not only normal hematopoiesis but also disease-specific alterations. Recent studies have combined immune cells, leukemic blasts, and adipocytes within a single OoC platform [132,133], and have provided insights into how leukemic cells exploit the BM microenvironment to evade immune attack and resist chemotherapeutic agents. These platforms allow for real-time tracking of CAR-T cell extravasation, leukemic cell recognition, and subsequent killing—all while under the influence of adipocyte-derived factors. Integrating patient-derived leukemic cells and adipocytes into bone marrow-on-chip platforms offers a route to personalized medicine. Such ex vivo models allow for the screening of multiple drug combinations and the prediction of individual responses. As previously highlighted [134], these systems can capture patient-specific variations and may guide treatment choices to improve clinical outcomes.

By bridging the gap between the limitations of animal models and the complexity of human disease, these innovative approaches hold the potential to unravel the metabolic and signaling networks that drive leukemia progression. They could also pave the way for novel therapeutic strategies, offering hope for more effective treatments in the fight against leukemia.

## Data Availability

No new data were created or analyzed in this study.
